# (*E*)-1-Phenyl-2-({5-[(1*E*)-(2-phenyl­hydrazin-1-yl­idene)meth­yl]-2-thien­yl}methyl­idene)hydrazine

**DOI:** 10.1107/S1600536810003302

**Published:** 2010-02-03

**Authors:** Geraldo M. de Lima, William T. A. Harrison, Edward R. T. Tiekink, James L. Wardell, Solange M. S. V. Wardell

**Affiliations:** a Departamento de Quimica, ICEx, Universidade Federal de Minas Gerais, 31270-901 Belo Horizonte, MG, Brazil; b Department of Chemistry, University of Aberdeen, Meston Walk, Old Aberdeen AB24 3UE, Scotland; c Department of Chemistry, University of Malaya, 50603 Kuala Lumpur, Malaysia; d Centro de Desenvolvimento Tecnológico em Saúde (CDTS), Fundação Oswaldo Cruz (FIOCRUZ), Casa Amarela, Campus de Manguinhos, Av. Brasil 4365, 21040-900 Rio de Janeiro, RJ, Brazil; e CHEMSOL, 1 Harcourt Road, Aberdeen AB15 5NY, Scotland

## Abstract

The title mol­ecule, C_18_H_16_N_4_S, adopts a U-shape with the aromatic groups lying *syn* and orientated in the same direction as the thio­phene S atom. The conformation about each of the C=N bonds is *E*. Overall, the mol­ecule is curved as seen in the dihedral angle of 30.26 (19)° formed between the terminal benzene rings. In the crystal, supra­molecular chains along the *c* axis are formed by a combination of N—H⋯N hydrogen bonds and N—H⋯π inter­actions.

## Related literature

For specific uses of 2-substituted-thio­phenes as materials, see: Michaleviciute *et al.* (2007 ▶, 2009 ▶); Kwon *et al.* (2009 ▶). For their specific uses as biological agents, see: Sonar & Crooks (2009 ▶); Mellado & Cortes (2009 ▶); Satyanarayana *et al.* (2008 ▶); Lourenço *et al.* (2007 ▶). For the preparation of hydrazones of thio­phene­carbaldehydes, see: Kwon, *et al.* (2009 ▶); Wardell *et al.* (2007 ▶); Vaysse & Pastour (1964 ▶); Novitskii *et al.* (1961 ▶). For related structures, see: Wardell *et al.* (2007 ▶, 2010 ▶); Ferreira *et al.* (2009 ▶); Nogueira *et al.* (2010 ▶); de Lima *et al.* (2010 ▶).
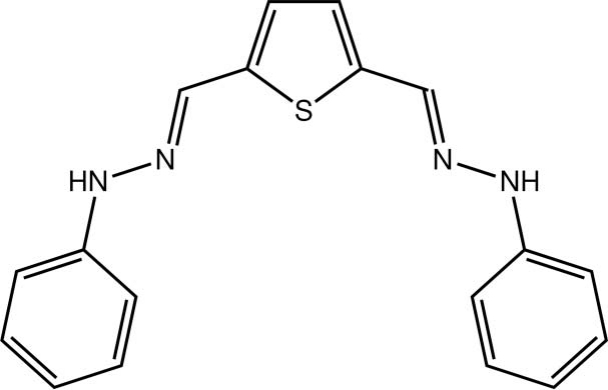


## Experimental

### 

#### Crystal data

C_18_H_16_N_4_S
*M* *_r_* = 320.41
Trigonal, 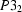

*a* = 15.6495 (6) Å
*c* = 5.9335 (10) Å
*V* = 1258.5 (2) Å^3^
*Z* = 3
Mo *K*α radiation
μ = 0.20 mm^−1^
*T* = 120 K
0.42 × 0.06 × 0.04 mm

#### Data collection

Nonius KappaCCD area-detector diffractometer
Absorption correction: multi-scan (*SADABS*; Sheldrick, 2007 ▶) *T*_min_ = 0.767, *T*_max_ = 1.000
11400 measured reflections
3675 independent reflections
3287 reflections with *I* > 2σ(*I*)
*R*_int_ = 0.059

#### Refinement

*R*[*F* ^2^ > 2σ(*F* ^2^)] = 0.050
*wR*(*F* ^2^) = 0.108
*S* = 1.04
3675 reflections
214 parameters
1 restraint
H atoms treated by a mixture of independent and constrained refinement
Δρ_max_ = 0.24 e Å^−3^
Δρ_min_ = −0.22 e Å^−3^
Absolute structure: Flack (1983 ▶), 1748 Friedel pairs
Flack parameter: 0.04 (10)

### 

Data collection: *COLLECT* (Hooft, 1998 ▶); cell refinement: *DENZO* (Otwinowski & Minor, 1997 ▶) and *COLLECT*; data reduction: *DENZO* and *COLLECT*; program(s) used to solve structure: *SHELXS97* (Sheldrick, 2008 ▶); program(s) used to refine structure: *SHELXL97* (Sheldrick, 2008 ▶); molecular graphics: *ORTEP-3* (Farrugia, 1997 ▶) and *DIAMOND* (Brandenburg, 2006 ▶); software used to prepare material for publication: *publCIF* (Westrip, 2010 ▶).

## Supplementary Material

Crystal structure: contains datablocks global, I. DOI: 10.1107/S1600536810003302/hg2637sup1.cif

Structure factors: contains datablocks I. DOI: 10.1107/S1600536810003302/hg2637Isup2.hkl

Additional supplementary materials:  crystallographic information; 3D view; checkCIF report

## Figures and Tables

**Table 1:** Hydrogen-bond geometry (Å, °) *Cg* is the centroid of the C6–C11 ring.

*D*—H⋯*A*	*D*—H	H⋯*A*	*D*⋯*A*	*D*—H⋯*A*
N2—H2n⋯N4^i^	0.88 (4)	2.58 (5)	3.398 (4)	155 (4)
C12—H12⋯N2^ii^	0.95	2.57	3.463 (5)	157
N4—H4*N*⋯*Cg*^ii^	0.89 (4)	2.81 (5)	3.415 (4)	126 (3)

Symmetry codes: (i) 
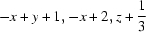
; (ii) 
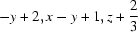
.

**Fig. 1.**
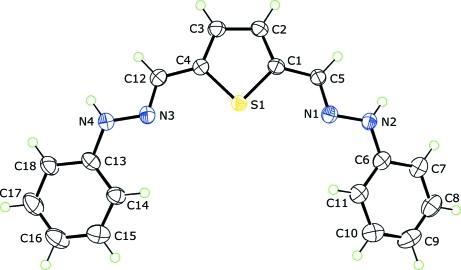
The molecular structure of (I) showing the atom-labelling scheme and displacement ellipsoids at the 50% probability level.

**Fig. 2.**
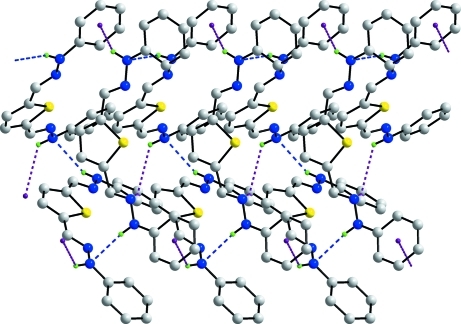
A view of the supramolecular chain in (I) mediated by N–H···N hydrogen bonds (blue dashed bonds) and N—H···π interactions (purple dashed lines). Hydrogen atoms not involved in these intermolecular interactions are omitted for reasons of clarity. Colour code: S, yellow; N, blue; C, grey; and H, green.

**Fig. 3.**
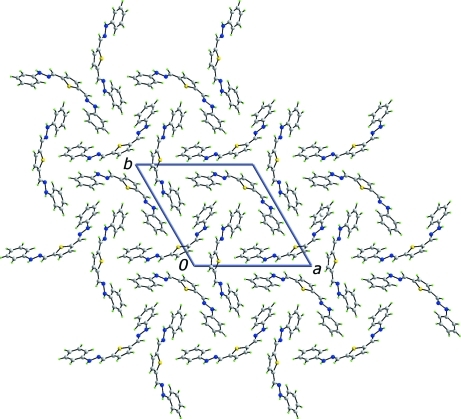
View in projection down the c axis of the unit cell contents for (I). Colour code: S, yellow; N, blue; C, grey; and H, green.

## supplementary crystallographic information

### Comment

Hydrazone and amide derivatives of thiophene have found many uses, for example in optoelectronic application (Michaleviciute *et al.*, 2007), as optical non-linear materials (Kwon *et al.*, 2009), as hole transporting materials (Michaleviciute *et al.*, 2009), and as biological agents (Sonar & Crooks, 2009; Mellado *et al.*, 2009; Satyanarayana *et al.*, 2008, Lourenço *et al.*, 2007). In continuation of structural studies on thiophene derivatives (Wardell *et al.*, 2007; Nogueira *et al.*, 2010; Ferreira *et al.*, 2009; Wardell *et al.*, 2010; de Lima *et al.*, 2010), we now report the structure of 2,5-thiophenedicarbaldehyde bis(phenylhydrazone), (I).

The molecule of (I) has a U-shaped conformation as the benzene rings are *syn*, lying to the same side of the molecule as the thiophene-S atom, Fig. 1. The conformation about each of the C5═N1 [1.282 (4) Å] and C12═N3 [1.287 (4) Å] double bonds is *E*. There are twists in the molecule, primarily about the hydrazine bonds, as seen in the values of the C5/N1/N2/C6 and C12/N3/N4/C13 torsion angles of -171.4 (3) and 165.1 (3) °. respectively. The dihedral angle formed between the two benzene rings is 30.26 (19) °.

Each of the hydrazine-N–H atoms participates in a significant intermolecular interactions to stabilise a supramolecular chain along the *c* axis, Fig. 2. The N2—H atom forms a conventional, albeit weak, N–H···N interaction, Table 1. The N4–H atom participates in a N–H···π interaction [N4–H···ring centroid(C6–C11)^i^ distance = 2.81 (5) Å, N4···ring centroid(C6–C11)^i^ = 3.415 (4) Å with an angle at H = 126 (3) ° for *i*: 2- y, 1+ x - y, 2/3+ z]. The resultant chain is further stabilised by C–H···N2 contacts, Table 1. The primary contacts between supramolecular chains are of the type C–H···π where the π-system is derived from the thiophene ring [C17–H···ring centroid(S1,C1–C4)^ii^ = 2.87 Å, C17······ring centroid(S1,C1–C4)^ii^ = 3.798 (6) Å, with angle at H = 165 ° for *ii*: 1-*x*+*y*, 1-*x*, -2/3+*z*], Fig. 3.

### Experimental

Solutions of phenylhydrazine.hydrochloride (0.22 g, 2 mmol) in EtOH (10 ml) and 2,5-thiophenedicarbaldehyde (0.14 g, 1 mmol) in EtOH (10 ml) were mixed. The reaction mixture was refluxed for 1 h, and rotary evaporated. The solid residue was recrystallised twice from aq. EtOH (v;v 1:2), m.p. 498-500 K. lit value 504 K (Vaysse & Pastour 1964) and 483-484 K (Novitskii *et al.*, 1961).

### Refinement

The C-bound H atoms were geometrically placed (C–H = 0.95 Å) and refined as riding with U_iso_(H) = 1.2U_eq_(C). The N-bound H atoms were located from a difference map and refined with U_iso_(H) = 1.5U_eq_(N).

### Crystal data

**Table:**

C_18_H_16_N_4_S	*D*_x_ = 1.268 Mg m^−^^3^
*M**_r_* = 320.41	Mo *K*α radiation, λ = 0.71073 Å
Trigonal, *P*3_2_	Cell parameters from 8754 reflections
Hall symbol: P 32	θ = 2.9–27.5°
*a* = 15.6495 (6) Å	µ = 0.20 mm^−^^1^
*c* = 5.9335 (10) Å	*T* = 120 K
*V* = 1258.5 (2) Å^3^	Rod, yellow
*Z* = 3	0.42 × 0.06 × 0.04 mm
*F*(000) = 504	

### Data collection

**Table:**

Nonius KappaCCD area-detector diffractometer	3675 independent reflections
Radiation source: Enraf Nonius FR591 rotating anode	3287 reflections with *I* > 2σ(*I*)
10 cm confocal mirrors	*R*_int_ = 0.059
Detector resolution: 9.091 pixels mm^-1^	θ_max_ = 27.5°, θ_min_ = 3.0°
φ and ω scans	*h* = −20→20
Absorption correction: multi-scan (*SADABS*; Sheldrick, 2007)	*k* = −20→20
*T*_min_ = 0.767, *T*_max_ = 1.000	*l* = −7→7
11400 measured reflections	

### Refinement

**Table:**

Refinement on *F*^2^	Secondary atom site location: difference Fourier map
Least-squares matrix: full	Hydrogen site location: inferred from neighbouring sites
*R*[*F*^2^ > 2σ(*F*^2^)] = 0.050	H atoms treated by a mixture of independent and constrained refinement
*wR*(*F*^2^) = 0.108	*w* = 1/[σ^2^(*F*_o_^2^) + (0.0104*P*)^2^ + 1.4032*P*] where *P* = (*F*_o_^2^ + 2*F*_c_^2^)/3
*S* = 1.04	(Δ/σ)_max_ < 0.001
3675 reflections	Δρ_max_ = 0.24 e Å^−^^3^
214 parameters	Δρ_min_ = −0.22 e Å^−^^3^
1 restraint	Absolute structure: Flack (1983), 1748 Friedel pairs
Primary atom site location: structure-invariant direct methods	Flack parameter: 0.04 (10)

### Special details

**Table:**

Geometry. All s.u.'s (except the s.u. in the dihedral angle between two l.s. planes) are estimated using the full covariance matrix. The cell s.u.'s are taken into account individually in the estimation of s.u.'s in distances, angles and torsion angles; correlations between s.u.'s in cell parameters are only used when they are defined by crystal symmetry. An approximate (isotropic) treatment of cell s.u.'s is used for estimating s.u.'s involving l.s. planes.
Refinement. Refinement of *F*^2^ against ALL reflections. The weighted *R*-factor wR and goodness of fit *S* are based on *F*^2^, conventional *R*-factors *R* are based on *F*, with *F* set to zero for negative *F*^2^. The threshold expression of *F*^2^ > 2σ(*F*^2^) is used only for calculating *R*-factors(gt) etc. and is not relevant to the choice of reflections for refinement. *R*-factors based on *F*^2^ are statistically about twice as large as those based on *F*, and *R*- factors based on ALL data will be even larger.

### Fractional atomic coordinates and isotropic or equivalent isotropic displacement parameters (Å^2^)

**Table:**

	*x*	*y*	*z*	*U*_iso_*/*U*_eq_	
S1	0.81560 (6)	0.77053 (6)	0.68924 (12)	0.02814 (16)	
N1	0.68935 (19)	0.86586 (19)	0.6256 (4)	0.0306 (6)	
N2	0.6286 (2)	0.9036 (2)	0.5913 (5)	0.0333 (6)	
H2N	0.614 (3)	0.927 (3)	0.711 (7)	0.050*	
N3	0.89915 (18)	0.63239 (18)	0.6731 (4)	0.0297 (6)	
N4	0.9375 (2)	0.5714 (2)	0.6555 (5)	0.0361 (6)	
H4N	0.963 (3)	0.563 (3)	0.782 (7)	0.054*	
C1	0.8031 (2)	0.8521 (2)	0.8654 (5)	0.0300 (7)	
C2	0.8520 (2)	0.8645 (3)	1.0644 (5)	0.0346 (7)	
H2	0.8543	0.9071	1.1812	0.041*	
C3	0.8988 (2)	0.8076 (3)	1.0785 (5)	0.0351 (7)	
H3	0.9360	0.8081	1.2055	0.042*	
C4	0.8851 (2)	0.7513 (2)	0.8902 (5)	0.0268 (6)	
C5	0.7407 (2)	0.8917 (2)	0.8070 (5)	0.0299 (6)	
H5	0.7374	0.9380	0.9049	0.036*	
C6	0.5620 (2)	0.8678 (2)	0.4142 (5)	0.0331 (7)	
C7	0.4833 (3)	0.8868 (3)	0.4091 (6)	0.0389 (8)	
H7	0.4741	0.9214	0.5294	0.047*	
C8	0.4193 (3)	0.8546 (3)	0.2278 (6)	0.0471 (9)	
H8	0.3660	0.8675	0.2242	0.056*	
C9	0.4316 (3)	0.8040 (3)	0.0517 (6)	0.0488 (10)	
H9	0.3871	0.7823	−0.0720	0.059*	
C10	0.5088 (3)	0.7852 (3)	0.0562 (6)	0.0431 (8)	
H10	0.5171	0.7502	−0.0647	0.052*	
C11	0.5747 (3)	0.8169 (2)	0.2359 (5)	0.0360 (7)	
H11	0.6279	0.8040	0.2374	0.043*	
C12	0.9187 (2)	0.6824 (2)	0.8568 (5)	0.0299 (6)	
H12	0.9559	0.6736	0.9714	0.036*	
C13	0.9022 (3)	0.4977 (2)	0.4896 (5)	0.0345 (7)	
C14	0.8457 (3)	0.4988 (2)	0.3078 (5)	0.0370 (7)	
H14	0.8257	0.5468	0.2990	0.044*	
C15	0.8196 (3)	0.4278 (3)	0.1403 (6)	0.0474 (9)	
H15	0.7811	0.4276	0.0162	0.057*	
C16	0.8481 (3)	0.3579 (3)	0.1506 (7)	0.0533 (11)	
H16	0.8311	0.3110	0.0328	0.064*	
C17	0.9020 (3)	0.3566 (3)	0.3340 (7)	0.0532 (11)	
H17	0.9202	0.3072	0.3437	0.064*	
C18	0.9296 (3)	0.4258 (2)	0.5029 (6)	0.0418 (8)	
H18	0.9671	0.4246	0.6275	0.050*	

### Atomic displacement parameters (Å^2^)

**Table:**

	*U*^11^	*U*^22^	*U*^33^	*U*^12^	*U*^13^	*U*^23^
S1	0.0322 (4)	0.0288 (4)	0.0255 (3)	0.0168 (4)	0.0005 (3)	−0.0002 (3)
N1	0.0320 (14)	0.0295 (14)	0.0300 (13)	0.0151 (12)	0.0019 (11)	0.0043 (10)
N2	0.0406 (16)	0.0391 (16)	0.0295 (13)	0.0268 (14)	−0.0022 (12)	−0.0007 (11)
N3	0.0242 (13)	0.0246 (13)	0.0388 (14)	0.0111 (11)	0.0010 (11)	0.0006 (11)
N4	0.0395 (16)	0.0385 (17)	0.0379 (15)	0.0252 (14)	−0.0053 (12)	−0.0063 (12)
C1	0.0312 (17)	0.0308 (16)	0.0274 (14)	0.0150 (14)	0.0016 (12)	−0.0010 (12)
C2	0.0378 (18)	0.0400 (19)	0.0304 (16)	0.0228 (16)	−0.0030 (13)	−0.0084 (13)
C3	0.0350 (18)	0.044 (2)	0.0298 (16)	0.0226 (16)	−0.0032 (13)	−0.0057 (14)
C4	0.0230 (14)	0.0280 (15)	0.0272 (14)	0.0112 (12)	0.0024 (11)	0.0020 (12)
C5	0.0342 (17)	0.0300 (16)	0.0283 (15)	0.0182 (14)	0.0002 (12)	−0.0003 (12)
C6	0.0317 (17)	0.0304 (17)	0.0319 (16)	0.0115 (14)	0.0039 (13)	0.0095 (12)
C7	0.0340 (18)	0.044 (2)	0.0395 (18)	0.0198 (16)	0.0056 (14)	0.0091 (15)
C8	0.0309 (19)	0.052 (2)	0.052 (2)	0.0162 (17)	−0.0004 (16)	0.0148 (18)
C9	0.037 (2)	0.050 (2)	0.042 (2)	0.0083 (18)	−0.0089 (16)	0.0092 (17)
C10	0.040 (2)	0.038 (2)	0.0389 (19)	0.0102 (16)	0.0000 (15)	0.0029 (15)
C11	0.0335 (17)	0.0313 (17)	0.0378 (17)	0.0122 (15)	0.0025 (13)	0.0062 (13)
C12	0.0244 (15)	0.0318 (16)	0.0313 (15)	0.0124 (13)	0.0015 (12)	0.0031 (12)
C13	0.0378 (18)	0.0277 (16)	0.0346 (16)	0.0137 (14)	0.0059 (14)	−0.0012 (13)
C14	0.0415 (19)	0.0304 (18)	0.0357 (17)	0.0154 (16)	−0.0010 (14)	−0.0015 (13)
C15	0.050 (2)	0.041 (2)	0.0405 (19)	0.0145 (19)	−0.0006 (17)	−0.0031 (16)
C16	0.069 (3)	0.033 (2)	0.050 (2)	0.020 (2)	0.002 (2)	−0.0097 (17)
C17	0.074 (3)	0.033 (2)	0.056 (2)	0.029 (2)	0.006 (2)	−0.0014 (17)
C18	0.056 (2)	0.0297 (17)	0.0424 (19)	0.0239 (17)	−0.0019 (17)	−0.0025 (15)

### Geometric parameters (Å, °)

**Table:**

S1—C4	1.738 (3)	C7—H7	0.9500
S1—C1	1.736 (3)	C8—C9	1.382 (6)
N1—C5	1.282 (4)	C8—H8	0.9500
N1—N2	1.361 (4)	C9—C10	1.379 (5)
N2—C6	1.386 (4)	C9—H9	0.9500
N2—H2N	0.88 (4)	C10—C11	1.391 (5)
N3—C12	1.287 (4)	C10—H10	0.9500
N3—N4	1.362 (4)	C11—H11	0.9500
N4—C13	1.402 (4)	C12—H12	0.9500
N4—H4N	0.89 (4)	C13—C18	1.392 (5)
C1—C2	1.368 (4)	C13—C14	1.400 (5)
C1—C5	1.436 (4)	C14—C15	1.391 (5)
C2—C3	1.410 (5)	C14—H14	0.9500
C2—H2	0.9500	C15—C16	1.374 (6)
C3—C4	1.371 (4)	C15—H15	0.9500
C3—H3	0.9500	C16—C17	1.383 (6)
C4—C12	1.431 (4)	C16—H16	0.9500
C5—H5	0.9500	C17—C18	1.377 (5)
C6—C11	1.396 (5)	C17—H17	0.9500
C6—C7	1.405 (5)	C18—H18	0.9500
C7—C8	1.382 (5)		
			
C4—S1—C1	91.41 (15)	C9—C8—H8	119.6
C5—N1—N2	116.9 (3)	C10—C9—C8	119.8 (3)
N1—N2—C6	119.0 (3)	C10—C9—H9	120.1
N1—N2—H2N	116 (3)	C8—C9—H9	120.1
C6—N2—H2N	119 (3)	C9—C10—C11	120.7 (4)
C12—N3—N4	115.9 (3)	C9—C10—H10	119.6
N3—N4—C13	120.0 (3)	C11—C10—H10	119.6
N3—N4—H4N	115 (3)	C10—C11—C6	119.6 (3)
C13—N4—H4N	119 (3)	C10—C11—H11	120.2
C2—C1—C5	126.9 (3)	C6—C11—H11	120.2
C2—C1—S1	111.3 (2)	N3—C12—C4	120.7 (3)
C5—C1—S1	121.6 (2)	N3—C12—H12	119.6
C1—C2—C3	113.1 (3)	C4—C12—H12	119.6
C1—C2—H2	123.5	C18—C13—C14	120.3 (3)
C3—C2—H2	123.5	C18—C13—N4	118.1 (3)
C4—C3—C2	113.2 (3)	C14—C13—N4	121.5 (3)
C4—C3—H3	123.4	C15—C14—C13	118.4 (3)
C2—C3—H3	123.4	C15—C14—H14	120.8
C3—C4—C12	126.7 (3)	C13—C14—H14	120.8
C3—C4—S1	111.0 (2)	C16—C15—C14	121.4 (4)
C12—C4—S1	122.2 (2)	C16—C15—H15	119.3
N1—C5—C1	121.4 (3)	C14—C15—H15	119.3
N1—C5—H5	119.3	C15—C16—C17	119.3 (4)
C1—C5—H5	119.3	C15—C16—H16	120.3
N2—C6—C11	120.8 (3)	C17—C16—H16	120.3
N2—C6—C7	119.5 (3)	C18—C17—C16	121.0 (4)
C11—C6—C7	119.6 (3)	C18—C17—H17	119.5
C8—C7—C6	119.5 (4)	C16—C17—H17	119.5
C8—C7—H7	120.2	C17—C18—C13	119.5 (4)
C6—C7—H7	120.2	C17—C18—H18	120.3
C7—C8—C9	120.9 (4)	C13—C18—H18	120.3
C7—C8—H8	119.6		
			
C5—N1—N2—C6	−171.4 (3)	C7—C8—C9—C10	0.0 (5)
C12—N3—N4—C13	165.1 (3)	C8—C9—C10—C11	−0.2 (5)
C4—S1—C1—C2	1.3 (3)	C9—C10—C11—C6	0.4 (5)
C4—S1—C1—C5	−173.5 (3)	N2—C6—C11—C10	−177.5 (3)
C5—C1—C2—C3	173.6 (3)	C7—C6—C11—C10	−0.3 (5)
S1—C1—C2—C3	−0.9 (4)	N4—N3—C12—C4	177.9 (3)
C1—C2—C3—C4	−0.3 (4)	C3—C4—C12—N3	178.5 (3)
C2—C3—C4—C12	−176.8 (3)	S1—C4—C12—N3	0.7 (4)
C2—C3—C4—S1	1.3 (4)	N3—N4—C13—C18	−168.3 (3)
C1—S1—C4—C3	−1.5 (3)	N3—N4—C13—C14	15.3 (5)
C1—S1—C4—C12	176.7 (3)	C18—C13—C14—C15	−1.2 (5)
N2—N1—C5—C1	176.6 (3)	N4—C13—C14—C15	175.2 (3)
C2—C1—C5—N1	−171.7 (3)	C13—C14—C15—C16	−0.1 (6)
S1—C1—C5—N1	2.2 (4)	C14—C15—C16—C17	1.6 (6)
N1—N2—C6—C11	−18.7 (4)	C15—C16—C17—C18	−1.8 (7)
N1—N2—C6—C7	164.1 (3)	C16—C17—C18—C13	0.5 (6)
N2—C6—C7—C8	177.3 (3)	C14—C13—C18—C17	1.0 (5)
C11—C6—C7—C8	0.1 (5)	N4—C13—C18—C17	−175.5 (3)
C6—C7—C8—C9	0.0 (5)		

### Hydrogen-bond geometry (Å, °)

**Table:**

Cg is the centroid of the C6–C11 ring.

**Table:**

*D*—H···*A*	*D*—H	H···*A*	*D*···*A*	*D*—H···*A*
N2—H2n···N4^i^	0.88 (4)	2.58 (5)	3.398 (4)	155 (4)
C12—H12···N2^ii^	0.95	2.57	3.463 (5)	157
N4—H4N···Cg^ii^	0.89 (4)	2.81 (5)	3.415 (4)	126 (3)

Symmetry codes: (i) −*x*+*y*+1, −*x*+2, *z*+1/3; (ii) −*y*+2, *x*−*y*+1, *z*+2/3.
